# Supplementary material for: Conjugation Operons in Gram-Positive Bacteria with and without Antitermination Systems
Source: Microorganisms. 2022 Mar 8;10(3):587. doi: 10.3390/microorganisms10030587 (PMC8955417; doi:10.3390/microorganisms10030587)
Supplement: Supplementary file 1 [file microorganisms-10-00587-s001.zip › microorganisms-1583695-supplementary.pdf]

microorganisms

MDPI

Supplementary materials

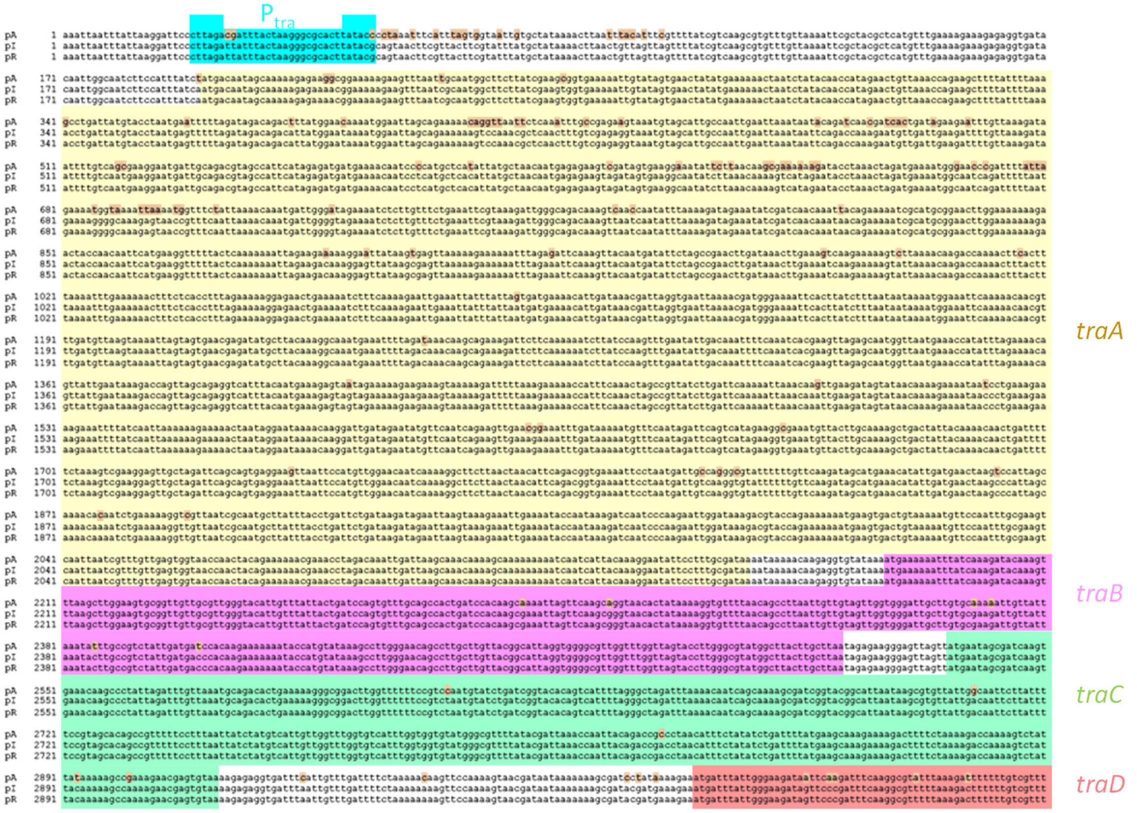

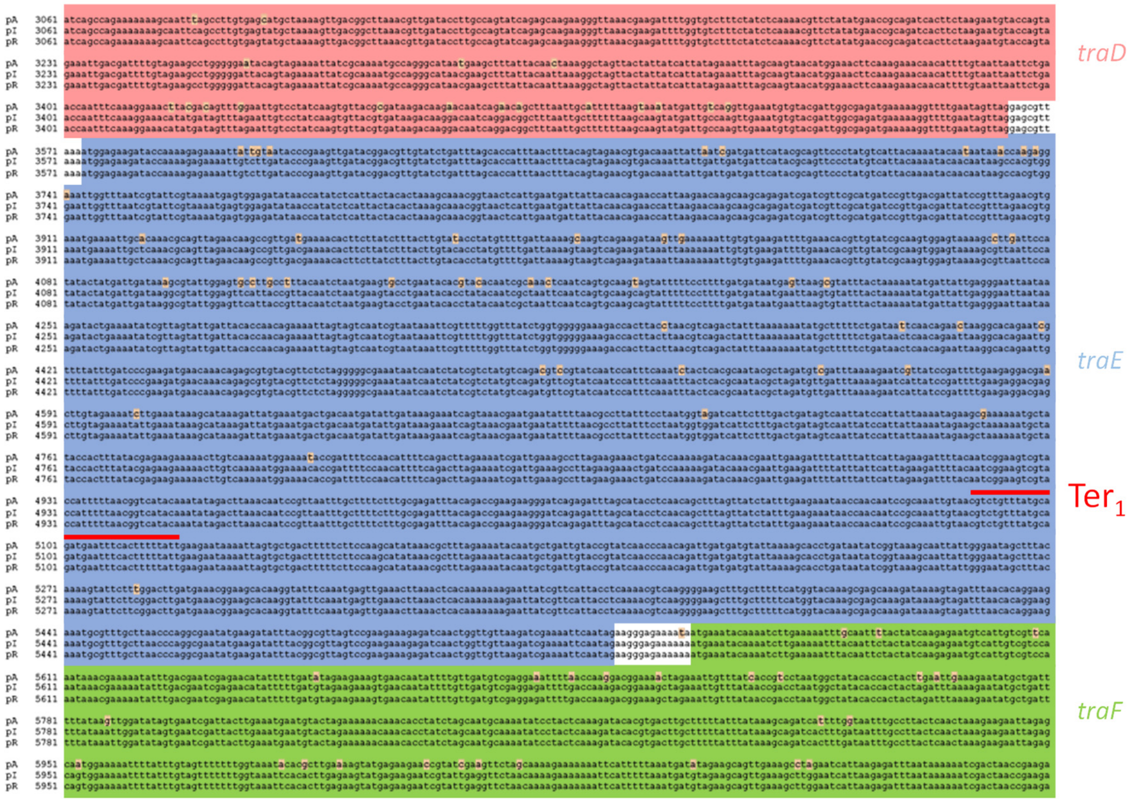

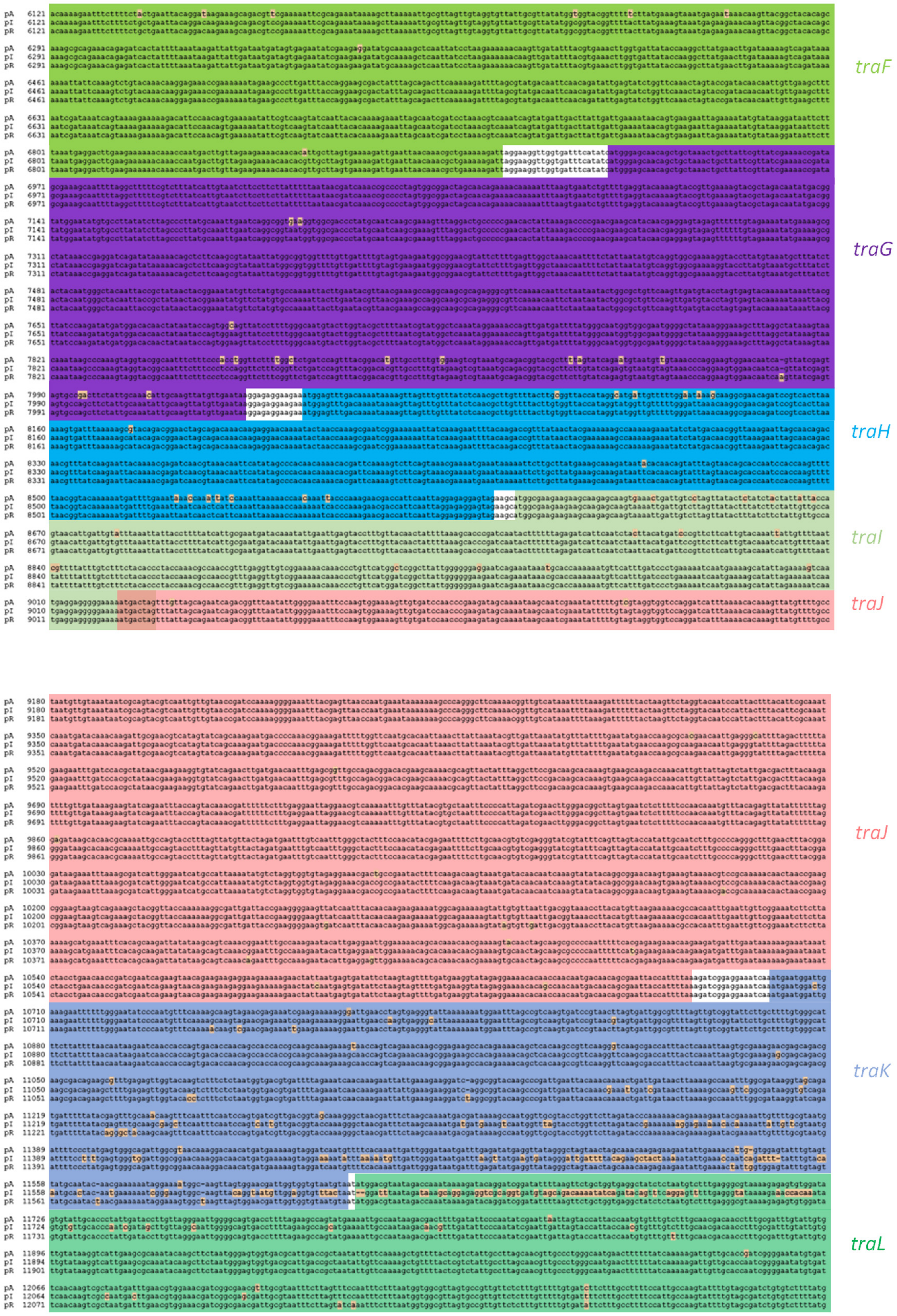
traF
traG
traH
traI
traJ
traJ
traK
traL

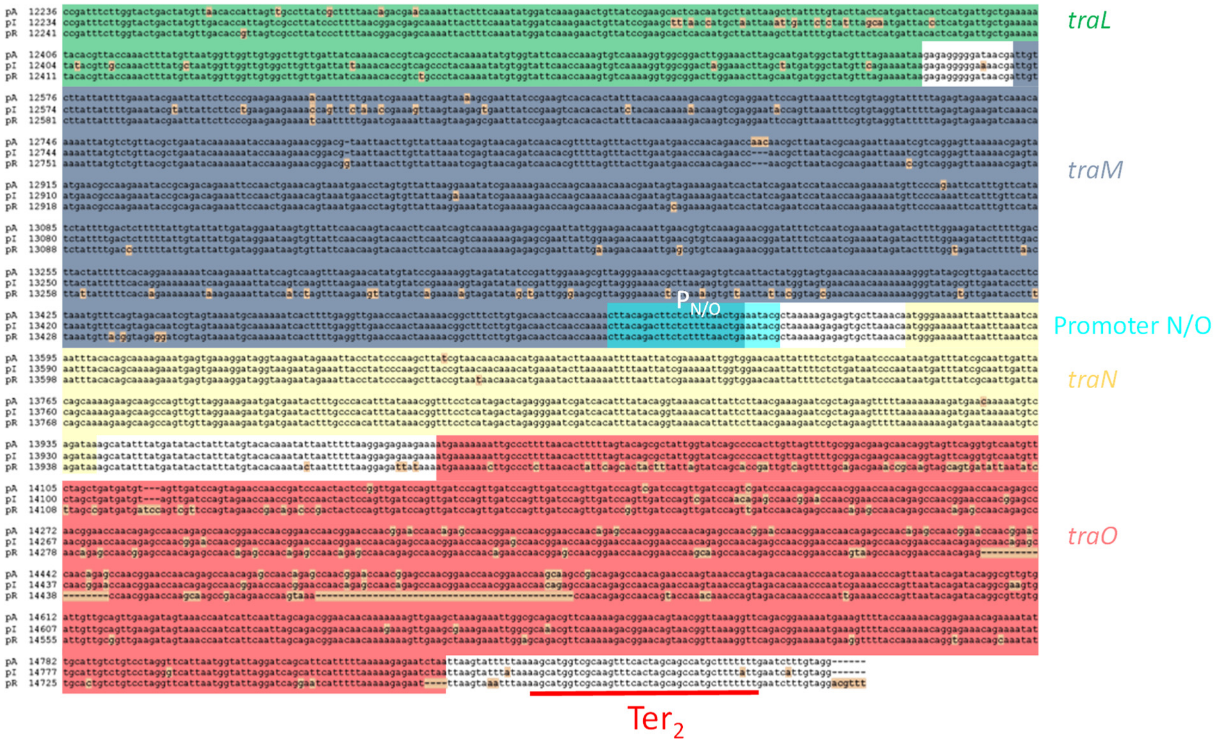

Figure S1. Alignment of the DNA sequences of the conjugation operons of Inc18 plasmids plP501, pAMβ1 and pRE25. Non conserved bp are shown on an orange background. The sequences of the conjugations operons including the upstream promoter and downstream Ter2 sequences are shown for plasmids pAMβ1 (pA), pIP501 (pI) and pRE25 (pR). Coding DNA sequences of the conjugation genes are indicated with colours and the name of the corresponding gene is given at the right. Positions of the identified putative terminators are indicated with a red line. Promoters Ptra and Pn are highlighted with a light blue colour.
